# Ketogenic Diet and Brain Health: Cerebrovascular Mechanisms, Neuroprotection, and Translational Implications

**DOI:** 10.3390/nu18071091

**Published:** 2026-03-29

**Authors:** Noémi Mózes, Ágnes Fehér, Tamás Csípő, Vince Fazekas-Pongor, Ágnes Lipécz, Dávid Major, Andrea Lehoczki, Norbert Dósa, Kata Pártos, Boglárka Csík, Hung Wei Yi, Csilla Kaposvári, Krisztián Horváth, Mónika Fekete

**Affiliations:** 1 Institute of Preventive Medicine and Public Health, Semmelweis University, 1089 Budapest, Hungary; mozes.noemi@semmelweis.hu (N.M.); trombitasne.feher.agnes@semmelweis.hu (Á.F.); csipo.tamas@semmelweis.hu (T.C.); pongor.vince@semmelweis.hu (V.F.-P.); lipecz.agnes@semmelweis.hu (Á.L.); major.david@semmelweis.hu (D.M.); ceglediandi@freemail.hu (A.L.); dosa.norbert@semmelweis.hu (N.D.); partos.katalin@semmelweis.hu (K.P.); csik.boglarka@semmelweis.hu (B.C.); hung.wei.yi@semmelweis.hu (H.W.Y.); kaposvari.csilla@semmelweis.hu (C.K.); horvath.krisztian@semmelweis.hu (K.H.); 2 Fodor Center for Prevention and Healthy Aging, Semmelweis University, 1085 Budapest, Hungary; 3 Health Sciences Division, Doctoral College, Semmelweis University, 1085 Budapest, Hungary

**Keywords:** ketogenic dietary therapies, cerebrovascular health, neuroprotection, brain aging, vascular aging, translational research

## Abstract

Background: Ketogenic dietary therapies (KDTs), characterized by substantial carbohydrate restriction and increased dietary fat intake, were originally developed for the treatment of drug-resistant epilepsy but have recently attracted broader scientific interest. In the context of population aging and the increasing prevalence of cognitive impairment and dementia, their potential relevance for brain health has received growing attention. Experimental and emerging clinical evidence suggests that ketogenic metabolism may influence biological processes involved in brain aging, including cerebrovascular regulation, neuroinflammatory signaling, and cerebral energy metabolism. Objective: This narrative review aims to synthesize current evidence on the relationship between ketogenic dietary therapies and brain health, with particular emphasis on cerebrovascular mechanisms, neuroinflammatory pathways, and neuroprotective processes relevant to aging. The review also briefly introduces the Semmelweis Study as an example of a translational research framework for evaluating nutrition-related interventions in real-world preventive settings. Methods: A narrative literature review was conducted using structured searches of major scientific databases to identify experimental and human studies investigating ketogenic dietary interventions, cerebrovascular mechanisms, and neuroprotective outcomes. Publications related to the Semmelweis Study were included solely to illustrate implementation-oriented research approaches and not as evidence supporting dietary efficacy. Results: Available evidence indicates that ketogenic dietary interventions may modulate several biological pathways relevant to brain health, including cerebral energy metabolism, mitochondrial function, oxidative stress regulation, and inflammatory signaling. However, the current evidence base is dominated by preclinical studies and short-term human investigations, and direct evidence linking ketogenic dietary therapies to long-term cerebrovascular or cognitive outcomes remains limited. Conclusions: Ketogenic dietary therapies represent metabolically distinct dietary strategies with potential relevance for cerebrovascular and neuroprotective mechanisms. Nevertheless, human evidence remains heterogeneous and insufficient to support broad clinical recommendations. Future research should prioritize well-designed long-term human studies with clearly defined metabolic, cerebrovascular, and cognitive endpoints. Translational research frameworks may facilitate the evaluation of feasibility, safety, and implementation of ketogenic interventions in aging populations.

## 1. Introduction

Nutrition is a fundamental component of lifestyle and a key determinant of health across the life course, making dietary quality and composition highly relevant for disease prevention and healthy aging [1]. Among dietary approaches that have attracted growing scientific attention in recent decades, ketogenic dietary therapies (KDTs) represent one of the most widely discussed strategies. Originally developed for the treatment of drug-resistant epilepsy, ketogenic dietary approaches have increasingly been investigated in metabolic, neurological, and preventive health contexts.

Although KDTs have been extensively studied in relation to metabolic disorders, their potential relevance for brain health—particularly through cerebrovascular and neuroinflammatory mechanisms—has not yet been coherently integrated within the existing literature [1,2,3]. Accumulating experimental and clinical evidence suggests that ketogenic metabolism may influence cerebral energy metabolism, vascular function, and inflammatory signaling pathways involved in brain aging [1,2,3,4,5]. However, current findings remain dispersed across multiple research domains, and the links between ketogenic interventions, cerebrovascular regulation, neurovascular coupling, and age-related vascular dysfunction are often interpreted inconsistently. An additional challenge in interpreting the available evidence is distinguishing ketosis-dependent biological effects from broader metabolic changes accompanying dietary interventions. Reported benefits of ketogenic diets may partly reflect caloric restriction, weight loss, improved glycemic control, or enhanced dietary adherence rather than ketosis-specific mechanisms alone. Consequently, careful interpretation of the literature requires distinguishing between ketosis-related mechanisms and broader metabolic effects associated with dietary modification.

In this review, metabolic conditions frequently discussed in relation to ketogenic dietary therapies—including obesity, type 2 diabetes, gut microbiota alterations, and longevity-related pathways—are considered only insofar as they influence cerebrovascular integrity, neuroinflammation, and neurovascular function. These factors are therefore addressed as interconnected contributors to the metabolic and vascular vulnerability of the aging brain rather than as independent domains. Accordingly, the present review focuses specifically on cerebrovascular and neuroprotective mechanisms potentially linking ketogenic metabolism to brain aging, rather than providing a comprehensive evaluation of ketogenic dietary approaches across all metabolic conditions.

Dietary quality plays a critical role in shaping health outcomes, yet achieving optimal nutrition has become increasingly challenging in modern societies. The accelerated pace of daily life has reduced the time available for food selection and preparation, contributing to the widespread consumption of ultra-processed foods [2,3]. These products—including sweets, fast food, and sugar-sweetened beverages—are typically energy-dense, highly palatable, and easily accessible but are associated with deterioration in overall dietary quality [4,5].

Ultra-processed foods are often characterized by high levels of added sugars, saturated and trans fats, and sodium, while being deficient in essential micronutrients, dietary fiber, and bioactive compounds such as antioxidants [6]. Diets dominated by such “empty-calorie” foods have been consistently linked to adverse metabolic outcomes, including obesity, cardiovascular disease, hypertension, and obesity-related cancers [7,8,9,10]. According to World Health Organization data from 2022, the global prevalence of adult obesity has more than doubled since 1990, while adolescent obesity has nearly quadrupled; approximately 43% of adults are classified as overweight and 16% as obese worldwide [7].

The public health burden associated with diet-related non-communicable diseases is substantial, both in terms of healthcare costs and long-term disability [9,11]. Poor dietary quality represents a modifiable risk factor strongly associated with increased cardiometabolic and cerebrovascular risk [12,13]. These effects are particularly relevant in the context of aging, as metabolic dysregulation and vascular dysfunction are key drivers of cognitive decline and neurodegenerative processes. As life expectancy continues to increase and a growing proportion of the population reaches advanced age, preserving both physical and cognitive function has become a major public health priority. Accordingly, dietary strategies that may support vascular health, cerebral metabolism, and neuroprotection warrant careful evaluation within the broader framework of healthy aging. Within this context, obesity and metabolic disease are considered primarily as vascular risk modifiers rather than primary endpoints of ketogenic dietary interventions.

## 2. Methods

This review was conducted as a structured narrative literature review aimed at synthesizing current evidence on the potential effects of ketogenic dietary therapies on cerebrovascular health, neuroprotection, and biological mechanisms relevant to brain aging. A narrative synthesis approach was selected because the available literature includes heterogeneous evidence from preclinical experiments, clinical intervention studies, and epidemiological observations that cannot be meaningfully integrated using formal quantitative meta-analytic methods.

### 2.1. Literature Search Strategy

A comprehensive literature search was performed using the electronic databases PubMed, Scopus, and Web of Science. The search included peer-reviewed publications available up to December 2025. No strict lower date limit was applied in order to capture seminal mechanistic and clinical studies relevant to ketogenic metabolism and brain function. Search strategies combined controlled vocabulary terms and keywords related to dietary exposure, mechanistic pathways, and clinical outcomes. Representative search terms included “ketogenic diet,” “ketogenic dietary therapy,” “low-carbohydrate diet,” “nutritional ketosis,” “β-hydroxybutyrate,” “cerebrovascular health,” “neurovascular function,” “blood–brain barrier,” “neuroinflammation,” “cognitive function,” “brain aging,” “mitochondrial function,” and “metabolic health.” These terms were combined using Boolean operators (AND/OR) to identify studies examining ketogenic metabolism and its potential cerebrovascular or neuroprotective implications. In addition to database searches, the reference lists of relevant review articles, systematic reviews, meta-analyses, and seminal studies were manually screened to identify additional publications of potential relevance.

### 2.2. Study Selection and Eligibility Criteria

Studies were considered eligible if they examined mechanistic, physiological, or clinical pathways through which ketogenic dietary interventions may influence cerebrovascular function, brain metabolism, or neuroprotective processes. Eligible publications included randomized controlled trials, clinical intervention studies, observational cohort studies, systematic reviews, meta-analyses, and mechanistic investigations conducted in relevant animal models. Particular attention was given to studies addressing biological mechanisms relevant to brain aging, including cerebral energy metabolism, glucose and insulin regulation, endothelial and vascular function, oxidative stress, neuroinflammation, mitochondrial bioenergetics, neurovascular coupling, and ketone body–mediated signaling pathways. Evidence related to metabolic and vascular risk factors frequently associated with aging—such as obesity, insulin resistance, and type 2 diabetes—was included only when these factors were directly linked to cerebrovascular integrity, neurovascular regulation, or brain energy metabolism.

### 2.3. Evidence Synthesis

Given the substantial heterogeneity in study designs, populations, and outcome measures across the available literature, the evidence was synthesized using a conceptual and mechanistic integration approach rather than quantitative pooling. Evidence from human clinical studies, observational research, and preclinical models was evaluated collectively in order to identify convergent biological pathways linking ketogenic metabolism with cerebrovascular regulation and neuroprotective mechanisms. When possible, findings were interpreted according to hierarchical levels of evidence. Priority was given to randomized controlled trials and systematic reviews, followed by observational studies and short-term intervention trials, while preclinical and mechanistic studies were considered primarily to support biological plausibility. This stratified interpretation aimed to minimize overgeneralization and to highlight areas where robust human evidence remains limited. Due to the heterogeneity of study designs, interventions, and reported outcomes, a formal quantitative meta-analysis was not performed.

### 2.4. Scope and Objectives of the Review

The scope of this review is deliberately focused on cerebrovascular and neuroprotective mechanisms relevant to brain aging. Consequently, the manuscript does not aim to provide a comprehensive evaluation of ketogenic dietary therapies in other domains such as weight management, sports performance, or general metabolic outcomes. Publications describing the Semmelweis Study and the Semmelweis Workplace Health Promotion Model Program were included solely to contextualize the translational and implementation perspective of nutrition-based research frameworks. These references should not be interpreted as evidence supporting the efficacy of ketogenic dietary interventions.

The primary objective of this narrative review is to synthesize and critically evaluate current evidence linking ketogenic dietary therapies with mechanisms relevant to cerebrovascular health, neuroprotection, and brain aging. Specifically, the review aims to do the following:

1. Describe the metabolic principles and physiological characteristics of ketogenic dietary therapies;
2. Summarize major ketogenic dietary variants and their typical clinical or experimental applications;
3. Evaluate mechanistic and clinical evidence connecting ketogenic metabolism with cerebrovascular function and neuroprotective processes;
4. Discuss findings related to aging and longevity within a vascular and metabolic framework;
5. Contextualize ketogenic dietary strategies within the translational framework of the Semmelweis Study; and
6. Critically examine the potential benefits, limitations, and safety considerations associated with ketogenic dietary interventions.

## 3. The Ketogenic Diet: Definition, Metabolic Basis, and Mechanistic Relevance

In dietary patterns dominated by refined carbohydrates, carbohydrate restriction represents one of the most direct nutritional strategies for improving metabolic regulation. Within this context, ketogenic dietary therapies have attracted increasing scientific interest as low-carbohydrate, high-fat dietary approaches capable of inducing a metabolic state characterized by nutritional ketosis [14,15]. KDTs are commonly characterized by substantial carbohydrate restriction, often below approximately 50 g/day in many nutritional protocols. However, classical therapeutic ketogenic diets are more precisely defined by a fat-to-non-fat ratio of approximately 3:1 or 4:1, which promotes sustained ketone body production and metabolic adaptation [16]. This metabolic transition results in increased circulating levels of acetoacetate, β-hydroxybutyrate, and acetone, collectively referred to as ketone bodies [17]. Following several days to weeks of sustained carbohydrate restriction, peripheral tissues and the central nervous system adapt to ketone utilization as an alternative energy substrate, a process commonly described as keto-adaptation [18].

Among ketone bodies, β-hydroxybutyrate (BHB) plays a central biological role. Unlike acetone, which is largely exhaled, and acetoacetate, which is rapidly interconverted, BHB readily crosses the blood–brain barrier and serves as an efficient oxidative substrate for neuronal mitochondria [19,20]. In brain energy metabolism, ketone bodies bypass several regulatory steps of glycolysis and enter the tricarboxylic acid cycle directly, which may enhance mitochondrial efficiency and reduce oxidative stress.

It is important to distinguish between nutritional ketosis, typically characterized by circulating BHB concentrations of approximately 0.5–3 mmol/L, and therapeutic ketosis, often achieved in classical ketogenic diet protocols used in epilepsy management, where ketone levels may be substantially higher. Many weight-loss–oriented ketogenic approaches produce only mild or moderate ketonemia and therefore differ metabolically from therapeutic ketogenic protocols. This neuroenergetic shift may be particularly relevant in the context of aging, where cerebrovascular dysfunction, impaired neurovascular coupling, endothelial alterations, and neuroinflammation frequently coexist with cerebral glucose hypometabolism. In addition, the early phase of ketogenic dietary interventions is often associated with depletion of hepatic and muscular glycogen stores, accompanied by transient water loss. This short-term effect has contributed to the perception of ketogenic diets primarily as rapid weight-loss strategies. Nevertheless, emerging evidence suggests that the potential relevance of ketogenic metabolism for brain health may extend beyond weight reduction and may involve vascular, inflammatory, and metabolic signaling pathways [19,20].

### 3.1. Ketogenic Diet Variants: Relevance Beyond Classification

Multiple ketogenic dietary therapy variants have been developed to improve feasibility and long-term adherence while maintaining nutritional ketosis [21]. Although detailed classification systems are well established, their relevance in the present review lies primarily in their mechanistic implications rather than in their exact dietary composition. Accordingly, a comparison of major ketogenic dietary therapy variants is summarized in Table 1, while the text focuses on mechanistically relevant distinctions.

From a neurovascular and neuroprotective perspective, ketogenic dietary therapies can be differentiated by their capacity to elevate circulating ketone levels, modulate insulin signaling, and influence lipid metabolism. Diets enriched in medium-chain triglycerides (MCTs), for example, promote relatively rapid ketone production compared with long-chain fat–based regimens, even at moderately higher carbohydrate intakes [26]. This feature may be relevant for brain energy metabolism because MCT-derived ketones can increase cerebral ketone availability without strict carbohydrate restriction. Similarly, less restrictive dietary models such as the modified Atkins diet (MAD) or low glycemic index treatment (LGIT) produce lower but more stable levels of ketosis and may exert milder metabolic effects while improving long-term adherence [27,28,32,33]. In contrast, very low-energy ketogenic therapies (VLEKT) induce marked short-term metabolic changes that are primarily associated with rapid weight loss and improvements in insulin sensitivity. However, concerns remain regarding their long-term sustainability and potential vascular implications, particularly in older populations [29,30,31].

### 3.2. Areas of Application: From Metabolic Control to Neurological Relevance

Ketogenic dietary therapies have been investigated across a broad range of clinical contexts, including obesity, epilepsy, type 2 diabetes, cardiovascular risk modulation, and neurological disorders. In metabolic conditions, carbohydrate restriction reduces postprandial glycemia and insulin secretion, thereby improving insulin sensitivity and promoting fatty-acid oxidation. These effects may be particularly relevant in aging populations, where insulin resistance and vascular dysfunction contribute to cognitive decline [30,31,32,33,34,35,36,37,38,39,40,41,42].

Historically, ketogenic dietary therapies were introduced as treatments for drug-resistant epilepsy, where sustained ketosis has been associated with reductions in seizure frequency through modulation of neuronal excitability and inhibitory neurotransmission [33,34,35,36,37,38]. Beyond epilepsy, increasing interest has emerged regarding the potential relevance of ketogenic metabolism in neurodegenerative disorders such as Alzheimer’s disease and Parkinson’s disease, conditions characterized by impaired cerebral glucose utilization and chronic neuroinflammation.

Evidence regarding cardiovascular health remains heterogeneous [43,44,45,46]. While some studies report improvements in triglycerides, HDL cholesterol, and blood pressure, responses of LDL cholesterol and apolipoprotein B (ApoB) are variable and clinically relevant for cardiovascular risk assessment. These inconsistencies highlight the importance of considering diet composition, intervention duration, and individual metabolic context when interpreting ketogenic dietary interventions [43,44,45,46,47,48,49].

In the context of physical performance, ketogenic diets shift substrate utilization toward fatty-acid oxidation, which may support endurance performance under certain physiological conditions. However, findings from randomized controlled trials remain inconsistent, and current evidence is insufficient to support generalized recommendations for athletic populations [50,51,52]. Finally, ketogenic dietary therapies have been explored as adjunctive metabolic strategies in oncology and inflammatory conditions, primarily based on hypotheses related to glucose restriction and immunometabolic modulation. Nevertheless, clinical evidence remains limited, and these approaches should currently be considered experimental rather than established therapeutic strategies [53,54,55,56,57,58,59].

## 4. Effect on Cerebrovascular Health and Neuroprotection

Ketogenic dietary therapies are well-established non-pharmacological interventions for drug-resistant epilepsy, with substantial clinical evidence supporting their efficacy, particularly in pediatric populations [60,61]. Beyond epilepsy, increasing interest has emerged regarding the potential relevance of ketogenic metabolic states in neurodegenerative and cerebrovascular disorders. This interest is largely driven by the potential influence of ketogenic metabolism on brain energy metabolism, mitochondrial function, and inflammatory signaling pathways [60,61]. Current knowledge derives from multiple sources, including human clinical studies, short-term intervention trials, observational investigations, and preclinical experimental models, each contributing differently to the evolving understanding of ketogenic metabolism and brain health.

In Alzheimer’s disease, impaired cerebral glucose utilization and insulin resistance are recognized as early pathophysiological features. Several studies have therefore explored whether ketogenic metabolism may partially compensate for this energetic deficit. For example, Reger et al. reported that medium-chain triglyceride (MCT) supplementation improved memory performance in patients with mild-to-moderate Alzheimer’s disease, with cognitive changes correlating with circulating β-hydroxybutyrate concentrations [62]. Nevertheless, human studies investigating ketogenic dietary therapies and cognitive outcomes remain relatively limited. Existing trials are generally characterized by small sample sizes, heterogeneous intervention protocols, relatively short intervention durations, and inconsistent reporting of dietary adherence and ketone-level verification [62,63,64].

Evidence regarding Parkinson’s disease is even more limited. A small uncontrolled clinical study reported short-term improvements in Unified Parkinson’s Disease Rating Scale scores following a 28-day ketogenic intervention [65]. However, the absence of a control group, together with the small sample size and short intervention duration, prevents causal conclusions. Observational findings suggesting associations between dietary fatty acid composition and Parkinson’s disease risk provide additional support for a potential metabolic contribution, but they do not establish a direct protective role for ketogenic dietary therapies [66]. Overall, these findings suggest that ketone bodies may provide an alternative cerebral energy substrate under conditions of impaired glucose metabolism. However, the current human evidence base remains limited and heterogeneous. Selected human studies examining the relationship between ketogenic dietary therapies and brain-related outcomes are summarized in Table 2.

From a cerebrovascular perspective, neurological injury following stroke, cerebral ischemia, or traumatic brain injury is largely driven by secondary injury cascades involving glutamate-mediated excitotoxicity, intracellular calcium overload, mitochondrial dysfunction, and excessive production of reactive oxygen species (ROS). These mechanisms overlap with pathways implicated in chronic neurodegenerative diseases and are closely related to key cerebrovascular processes, including neurovascular unit integrity, blood–brain barrier function, cerebrovascular reactivity, and neurovascular coupling. Experimental studies suggest that β-hydroxybutyrate may mitigate neuronal energy failure by preserving ATP levels, improving mitochondrial efficiency, and reducing oxidative stress, thereby potentially limiting neuronal injury. Additional ketone bodies, such as acetoacetate and acetone, have demonstrated similar neuroprotective properties in experimental models [70]. However, most of these findings derive from preclinical studies, and their clinical relevance in human cerebrovascular disease remains uncertain.

KDTs may also influence cerebrovascular risk indirectly through improvements in metabolic parameters, including insulin sensitivity and glycemic control. Given the strong association between diabetes, metabolic syndrome, and cerebrovascular disease, such metabolic effects could theoretically contribute to improved vascular brain health [71]. Some reported benefits may also reflect broader metabolic improvements accompanying dietary modification rather than ketosis-specific mechanisms alone.

Inflammation represents another mechanistic link between ketogenic metabolism and neuroprotection. Chronic low-grade inflammation contributes to both neurodegeneration and vascular aging. Ketosis has been shown to modulate inflammatory signaling pathways, including inhibition of NF-κB activation and reductions in pro-inflammatory cytokine production, largely through β-hydroxybutyrate–mediated mechanisms [72]. While these anti-inflammatory effects are consistently observed in experimental systems, their clinical significance in long-term human interventions remains uncertain.

**Figure 1:**
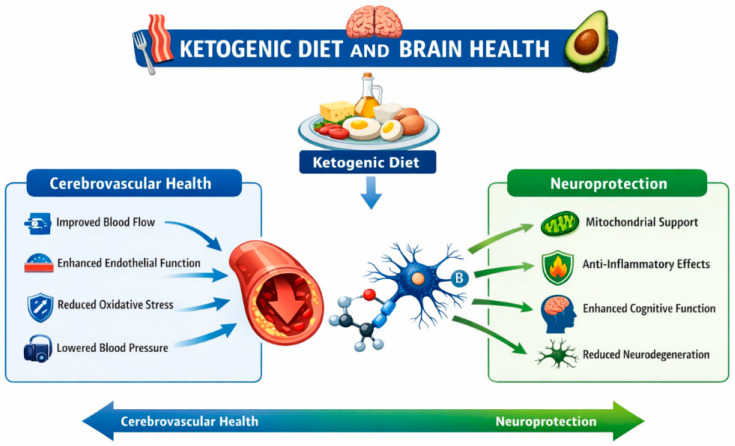
Proposed mechanistic pathways linking ketogenic metabolism with cerebrovascular regulation and neuroprotective processes. Ketogenic dietary therapies may influence cerebral energy metabolism, mitochondrial function, oxidative stress, inflammatory signaling, and vascular regulation. Evidence supporting these mechanisms derives predominantly from preclinical models and short-term human studies, and their long-term clinical relevance remains under investigation.

Overall, the majority of evidence supporting the potential neuroprotective and cerebrovascular effects of ketogenic dietary therapies derives from preclinical models and short-term human studies, which limits causal interpretation. Many human investigations involve relatively small sample sizes, lack appropriate control groups, or rely on surrogate metabolic outcomes rather than clinically meaningful cerebrovascular endpoints, such as neurovascular coupling, blood–brain barrier integrity, or measures of cerebrovascular reactivity. Consequently, current findings should be interpreted primarily as hypothesis-generating rather than definitive evidence. Future research should prioritize well-designed randomized controlled trials evaluating the long-term effects of ketogenic dietary therapies on cerebrovascular function, cognitive outcomes, and neurodegenerative disease progression. Improved mechanistic understanding may also support the development of alternative therapeutic strategies, including exogenous ketone supplementation or ketone precursors, which could potentially provide metabolic benefits without the challenges associated with long-term adherence to strict dietary regimens. Figure 1 summarizes the proposed mechanistic pathways through which ketogenic metabolism may influence cerebrovascular regulation and neuroprotective processes.

## 5. Ketogenic Diet and Longevity

The potential effects of ketogenic dietary therapies on longevity have been investigated primarily in preclinical models, with the majority of available evidence derived from murine studies [73,74,75,76]. Several experimental investigations have reported modest but statistically significant extensions of lifespan in mice maintained on ketogenic diets. For example, Newman et al. demonstrated that mice receiving a ketogenic diet exhibited an increase in median lifespan compared with animals fed standard chow, accompanied by improvements in age-related memory performance and selected composite health measures [73]. Similarly, Roberts et al. reported increased median survival and preservation of several physiological functions during aging in mice maintained on ketogenic diets. These effects were associated with tissue-specific modulation of mTORC1 signaling and increased protein acetylation, suggesting adaptive metabolic responses rather than uniform systemic effects [74].

Long-term metabolic consequences of ketogenic feeding have also been characterized in animal models. Douris et al. observed that mice fed a ketogenic diet exhibited sustained reductions in body weight and fat mass, increased energy expenditure, improved glucose homeostasis, and elevated circulating β-hydroxybutyrate concentrations [75]. These changes were accompanied by altered hepatic expression of key metabolic regulators, including increased fibroblast growth factor 21 (FGF21) and decreased expression of lipogenic enzymes. Metabolomic analyses further suggested adaptive shifts in amino acid metabolism, indicating that mice maintained on ketogenic diets may preserve amino acid balance despite prolonged carbohydrate restriction [75].

Additional studies initiating ketogenic feeding in late midlife have reported improvements in selected functional outcomes, including spatial memory and neuromuscular performance, in aged male mice [76]. Importantly, these findings predominantly reflect improvements in healthspan-related parameters rather than unequivocal extensions of maximal lifespan.

While ketogenic dietary interventions appear to influence metabolic pathways implicated in aging—including mitochondrial function, oxidative stress regulation, and nutrient-sensing signaling—their effects on longevity remain modest and context-dependent in animal models. Moreover, lifespan extensions observed in rodents cannot be directly extrapolated to humans, as murine ketogenic diets often differ substantially in protein composition, metabolic context, and feeding patterns compared with human dietary interventions.

To date, no large-scale, long-duration randomized controlled trials have evaluated the impact of ketogenic dietary therapies on human lifespan, and evidence regarding their potential influence on healthspan in aging populations remains limited and heterogeneous. Consequently, claims regarding longevity benefits of ketogenic interventions in humans should be interpreted with caution. Future research should prioritize well-designed longitudinal human studies that clearly distinguish between lifespan and healthspan outcomes while carefully evaluating the long-term safety, feasibility, and sustainability of ketogenic dietary patterns in aging populations.

## 6. Pros and Cons of the Ketogenic Diet

### 6.1. Potential Benefits of the Ketogenic Diet

The popularity of ketogenic dietary therapies has increased in recent years as physicians and researchers continue to explore their potential metabolic and neurological effects. Achieving nutritional ketosis, the metabolic state typically targeted by KDTs, involves substantial restriction of carbohydrate intake, moderate protein consumption, and an increased proportion of energy derived from dietary fats [73,74,77,78,79]. Under such conditions, carbohydrate restriction promotes a metabolic shift from glucose utilization toward fatty acid oxidation and the hepatic production of ketone bodies [80,81,82,83,84,85,86,87,88,89].

During ketosis, circulating ketone bodies—primarily β-hydroxybutyrate, acetoacetate, and acetone—can serve as alternative energy substrates for tissues with high metabolic demands, including the brain and skeletal muscle [90,91]. In addition to their energetic role, ketone bodies—particularly β-hydroxybutyrate—have been suggested to participate in cellular signaling processes related to appetite regulation and metabolic adaptation, although the clinical relevance of these mechanisms remains under investigation [90,91,92].

Studies examining low-carbohydrate, high-fat (LCHF) dietary patterns, including ketogenic dietary interventions, have reported short- to medium-term improvements in several metabolic outcomes. These include reductions in body weight, improvements in markers associated with metabolic syndrome, and enhanced glycemic control in individuals with type 2 diabetes [80,81,82]. Additional mechanistic and observational studies suggest that ketogenic metabolic states may influence inflammatory pathways, epigenetic regulation, gut microbiome composition, and lipid metabolism; however, the strength and consistency of these effects vary across studies and remain an active area of investigation [83].

The public health relevance of dietary strategies targeting metabolic regulation is underscored by the increasing prevalence of obesity, diabetes, and metabolic syndrome in the European Union and in Hungary [84,85]. These conditions represent major risk factors for cardiovascular disease, stroke, and neurodegenerative disorders such as Alzheimer’s disease [86]. Currently, approximately 60 million individuals live with diabetes in the European Region, representing about 10.3% of men and 9.6% of women aged 25 years and older. The prevalence of diabetes continues to rise across all age groups, largely driven by increasing rates of overweight and obesity, unhealthy dietary habits, and physical inactivity [87].

Because insulin resistance and carbohydrate intolerance contribute to the development of metabolic disorders, dietary strategies that reduce carbohydrate intake have been proposed as potential approaches to improve metabolic regulation [81]. Ketogenic dietary therapies encompass a spectrum of interventions that differ in their degree of carbohydrate restriction and clinical application. Strict therapeutic ketogenic diets are most commonly used in the management of drug-resistant epilepsy, while modified ketogenic approaches are increasingly investigated in metabolic and preventive health contexts [80].

From a metabolic perspective, carbohydrate restriction lowers circulating glucose concentrations and reduces insulin secretion, thereby promoting fatty acid oxidation and ketone production [88,89]. When hepatic fatty acid oxidation predominates, ketogenesis leads to the production of ketone bodies that can partially substitute for glucose as energy substrates in several tissues [90,91,92]. These metabolic adaptations form the physiological basis for the proposed therapeutic and metabolic effects of ketogenic dietary therapies.

### 6.2. Gut Microbiome Interactions and Implications for Ketogenic Dietary Therapies

Recent research indicates that gut microbiome composition is strongly influenced by lifestyle-related factors, including sleep patterns, physical activity, antibiotic exposure, and dietary habits [93,94,95]. According to Rothschild et al., the average heritability of gut microbiome taxa is relatively low (approximately 1.9%), whereas more than 20% of interindividual variability can be attributed to diet and lifestyle factors [96]. Consequently, increasing attention has been directed toward understanding the complex interactions between dietary patterns, gut microbial communities, and host metabolic processes.

Dietary composition plays a particularly important role in shaping microbiome diversity and metabolic activity. Studies examining prebiotic-rich foods, such as inulin and oligosaccharides, have demonstrated increases in Bifidobacteria abundance and other beneficial microbial taxa in the colon [97]. In contrast, Westernized dietary patterns characterized by high intake of processed foods and refined carbohydrates have been associated with reduced microbial diversity and unfavorable shifts in microbial composition [98]. For example, individuals consuming Westernized diets have been reported to exhibit increased Firmicutes and decreased Bacteroidetes abundance, microbial patterns that have been associated with adverse metabolic outcomes [98]. Similarly, dietary patterns rich in fruits, vegetables, and dietary fiber have been associated with increased microbiota diversity and improved metabolic health indicators [99].

Within this context, it is important to examine how dietary strategies such as ketogenic dietary therapies (KDTs) may influence gut microbiome diversity and microbial composition. Several studies suggest that whole grains and fiber-rich foods play a central role in maintaining microbiome stability and metabolic health [100,101,102,103]. Because strict ketogenic dietary patterns substantially limit carbohydrate intake, individuals following these diets may consume lower amounts of whole grains and certain fiber-rich foods, potentially affecting microbial diversity [104,105,106,107]. Adam-Perrot et al. reported that low-carbohydrate diets may be associated with an increased risk of nutritional inadequacy, particularly with respect to dietary fiber, vitamins, minerals, and iron intake [104]. Consequently, ketogenic dietary interventions should emphasize the inclusion of low-carbohydrate foods rich in dietary fiber, such as non-starchy vegetables, seeds, and nuts. In addition, appropriate protein intake—approximately 1.5 g/kg body weight per day—has been suggested in some ketogenic dietary protocols to support metabolic balance [108].

At present, long-term human data on the effects of ketogenic dietary therapies on gut microbiome composition remain limited. Some studies suggest that ketogenic interventions may influence microbial communities by increasing Bacteroidetes and Bifidobacteria abundance—bacterial taxa that have been associated with improved metabolic profiles—while reducing microbial species linked to metabolic dysfunction [109,110]. A lower Firmicutes/Bacteroidetes ratio has been proposed as a potential indicator of a healthier microbiome composition [111]. In contrast, individuals with obesity have been reported to exhibit higher Firmicutes/Bacteroidetes ratios and increased levels of short-chain fatty acids in stool samples [111,112]. Therefore, improvements in metabolic health and weight reduction observed during ketogenic interventions may indirectly contribute to favorable microbiome changes.

Further insights into diet–microbiome interactions were reported by Basciani et al., who examined gut microbiota changes in obese, insulin-resistant individuals following isocaloric ketogenic diets differing in protein source [113]. After 45 days of intervention, all very low-calorie ketogenic diets reduced Firmicutes abundance and increased Bacteroidetes levels. However, these favorable microbial shifts were less pronounced in participants consuming predominantly animal-derived protein sources, suggesting that dietary composition within ketogenic interventions may influence microbiome outcomes [113].

### 6.3. Long-Term Effects of the Ketogenic Diet and Its Potential Role in Obesity Management

The global rise in obesity represents a major public health challenge in developed countries, and numerous studies have examined the effects of different dietary strategies on body weight regulation and metabolic health. One commonly investigated approach involves modifying dietary macronutrient composition in order to reduce blood glucose concentrations and regulate insulin secretion. Carbohydrate restriction has been shown to lower circulating glucose and insulin levels in several clinical studies [114,115,116]. However, long-term adherence remains a critical determinant of success across all dietary interventions.

In a two-year randomized study conducted by Shai et al. [117], 322 moderately obese participants were assigned to one of three dietary interventions: a low-fat calorie-restricted diet, a Mediterranean calorie-restricted diet, or a low-carbohydrate non-calorie-restricted diet. More than 85% of participants remained adherent to their assigned dietary intervention throughout the study period. Individuals in the low-carbohydrate group followed a ketogenic dietary phase during the first two months, after which carbohydrate intake was gradually increased. This group initially experienced the greatest weight loss; however, body weight trajectories later converged toward those observed in the Mediterranean diet group after partial carbohydrate reintroduction. Importantly, the low-carbohydrate group achieved a comparable caloric deficit despite the absence of prescribed caloric restriction, suggesting that dietary composition may influence satiety and spontaneous energy intake.

Another intervention study involving 66 obese individuals (BMI > 30) instructed participants to consume fewer than 20 g of carbohydrates per day for 12 weeks, followed by a gradual increase to approximately 40 g/day [118]. Participants experienced significant reductions in body weight and BMI while maintaining nutritional ketosis. Continued weight reduction was observed during the intervention period, in contrast to the attenuation of weight loss observed in the Shai study following carbohydrate reintroduction.

One practical advantage of ketogenic dietary interventions is the availability of a measurable biomarker of adherence, β-hydroxybutyrate (BHB), with circulating concentrations above approximately 0.5 mmol/L commonly used as an indicator of nutritional ketosis. Mohorko et al. [116] reported that obese individuals maintaining elevated BHB levels during a 12-week intervention experienced significant weight reduction, primarily attributable to loss of fat mass. Similarly, in another clinical study, 92% of diabetic patients following a ketogenic dietary intervention achieved significant weight loss while maintaining ketosis, whereas participants receiving standard treatment did not demonstrate comparable changes [119].

Short-term ketogenic dietary interventions have also reported measurable metabolic changes. In a study involving obese Chinese women, participants followed their habitual diet for four weeks and subsequently adopted a ketogenic diet for an additional four weeks with equivalent caloric intake but carbohydrate intake below 10% of total energy [120]. Significant reductions were observed in body weight, BMI, waist and hip circumference, body fat percentage, and fasting leptin concentrations. Comparable findings have been reported in other ketogenic dietary studies [29,115].

Appetite regulation represents an important factor influencing long-term weight loss success. Castro et al. [121] observed a negative correlation between circulating BHB concentrations and subjective measures of appetite and hunger during periods of maximal ketosis, despite minimal changes in ghrelin concentrations. These findings are consistent with other studies suggesting that low-carbohydrate dietary patterns may influence hunger regulation and satiety differently from low-fat diets [122,123]. Nevertheless, it remains difficult to determine the relative contribution of ketosis-specific metabolic signaling versus broader metabolic changes such as caloric deficit or weight loss. Overall, available evidence suggests that ketogenic dietary strategies may contribute to weight reduction and metabolic improvements in certain populations, particularly in the short to medium term. However, long-term outcomes appear to depend on factors such as dietary adherence, total energy intake, and individual metabolic variability.

### 6.4. The Efficacy of Low-Carbohydrate Ketogenic Diet in Diabetes Management

Low-carbohydrate dietary strategies have historically been used in the management of diabetes, even prior to the discovery of insulin therapy. Carbohydrate restriction can reduce postprandial glucose excursions and improve glycemic control, including in individuals without marked overweight or obesity. In recent years, ketogenic dietary therapies (KDTs) have been investigated as a potential dietary strategy for improving metabolic regulation in patients with type 2 diabetes [124].

Among these approaches, very-low-energy ketogenic therapy (VLEKT) has been studied in the context of obesity-associated diabetes. Clinical studies suggest that VLEKT interventions may lead to significant reductions in blood glucose concentrations and improvements in glycemic control, facilitating achievement of therapeutic targets in selected patient populations [124].

In a clinical study by Westman et al., individuals with type 2 diabetes following a ketogenic dietary intervention demonstrated reductions in HbA1c from an average of 7.5% to 5.9% over a 15-month period, with several participants achieving substantial improvements in glycemic control [125]. Similarly, meta-analyses suggest that adherence to low-carbohydrate dietary patterns for at least six months may be associated with improvements in glycemic parameters and, in some cases, remission of type 2 diabetes under appropriate clinical supervision [126]. Additional studies have reported improvements in glycemic profiles and reductions in fasting glucose levels during ketogenic dietary interventions [127,128].

Although randomized controlled trials remain relatively limited, several smaller studies and case series have reported favorable metabolic outcomes [114,129]. Walton et al. described 11 case studies of individuals with type 2 diabetes consuming fewer than 30 g of carbohydrates per day, with reductions in HbA1c from values above 6.5% to approximately 5.6% [46]. Lichtash et al. reported a case of a normal-weight woman with type 2 diabetes whose HbA1c decreased from 9.3% to 5.8% following adoption of a ketogenic dietary pattern [130]. Wong et al. also observed improvements in glycemic control and reductions in medication requirements in patients with type 1 and type 2 diabetes adhering to a ketogenic dietary intervention for more than three months [129].

Importantly, reductions in antidiabetic medication during ketogenic dietary interventions require careful medical supervision, as rapid improvements in glycemic control may increase the risk of hypoglycemia or, in specific clinical contexts, euglycemic ketoacidosis. Furthermore, it remains difficult to determine the relative contribution of ketosis-specific versus broader metabolic mechanisms, such as caloric deficit, weight reduction, or improved dietary adherence. Overall, current evidence suggests that ketogenic dietary strategies may improve glycemic control in selected individuals with type 2 diabetes, particularly in the short to medium term. However, long-term randomized controlled trials are still required to clarify their durability, safety, and comparative effectiveness relative to other dietary interventions.

### 6.5. The Effect of Ketogenic Dietary Therapies on Cardiovascular Health

Cardiovascular disease remains one of the leading causes of morbidity and mortality worldwide [131]. Historically, high-fat dietary patterns were considered detrimental to cardiovascular health, which led to dietary recommendations limiting saturated fat intake to less than 10% of total energy intake [132]. However, recent evidence suggests that the relationship between dietary fat intake and cardiovascular risk is more complex than previously assumed.

Randomized controlled trials and epidemiological studies have questioned the use of total cholesterol and LDL cholesterol as sole predictors of cardiovascular risk, emphasizing the importance of a broader lipid and metabolic profile [133]. Alternative biomarkers, including apolipoprotein B (ApoB), the total cholesterol/HDL cholesterol ratio, small dense LDL particles, and the ApoB/ApoA1 ratio, have been proposed as more informative indicators of cardiovascular risk in certain populations [134].

Large prospective studies have also highlighted the role of dietary carbohydrate quality in cardiovascular health. For example, the EPIC study reported that dietary patterns characterized by high glycemic load and glycemic index were associated with an increased risk of coronary heart disease [135]. Similarly, the PURE study suggested that replacement of refined carbohydrates with dietary fats may be associated with improved cardiometabolic outcomes in some populations [136].

Nevertheless, the cardiovascular effects of ketogenic dietary therapies remain heterogeneous. While several studies report reductions in triglycerides and increases in HDL cholesterol, responses of LDL cholesterol and ApoB concentrations vary considerably between individuals and may depend on dietary fat composition, baseline metabolic status, and duration of the intervention. Consequently, the cardiovascular implications of ketogenic dietary strategies should be interpreted cautiously and evaluated within the broader context of overall dietary quality and individual metabolic risk profiles.

### 6.6. Ketogenic Dietary Therapies and Potential Implications for Cancer Metabolism

The relationship between nutrition and cancer development is complex and multifactorial. Chronic hyperinsulinemia and insulin resistance have been implicated in tumor progression and cancer-related metabolic dysregulation [137,138]. Within this context, ketogenic dietary therapies have been investigated as a potential metabolic strategy aimed at reducing circulating glucose levels and insulin signaling.

Experimental studies suggest that certain cancer cells exhibit reduced capacity to utilize ketone bodies due to impaired ketolytic enzyme activity, which has led to the hypothesis that carbohydrate restriction may create a metabolic environment less favorable for tumor growth [139]. However, most evidence supporting this concept derives from preclinical studies or small clinical investigations.

At present, ketogenic dietary therapies should be considered experimental adjunctive strategies rather than established cancer treatments. Clinical evidence remains limited, and nutritional management in oncology requires careful individualization, particularly in patients experiencing cancer-related metabolic disturbances such as tumor cachexia.

### 6.7. Risks and Potential Adverse Effects of Ketogenic Dietary Therapies

Dietary therapies are characterized by sustained restriction of carbohydrate intake combined with increased dietary fat consumption and moderate protein intake in order to induce nutritional ketosis [140]. Although these dietary approaches may provide metabolic benefits in certain clinical contexts, they are also associated with potential risks and may not be appropriate for all individuals [83].

Under normal physiological conditions, carbohydrates serve as the primary energy source for many tissues [141,142,143,144]. Severe carbohydrate restriction (typically below approximately 50 g/day) reduces insulin secretion and shifts metabolism toward increased lipolysis, gluconeogenesis, and ketone body production [145,146]. During nutritional ketosis, ketone bodies—including acetoacetate, β-hydroxybutyrate, and acetone—can partially replace glucose as metabolic substrates for several tissues, including the brain [147,148,149].

Although this metabolic adaptation may provide an alternative energy source, some studies suggest that alterations in brain energy metabolism under very low-carbohydrate conditions may influence cognitive performance in certain contexts [150,151]. Additional adverse effects associated with ketogenic dietary interventions include potential micronutrient deficiencies, gastrointestinal disturbances, nephrolithiasis, and unfavorable lipid responses in susceptible individuals [140].

Short-term side effects, often described as the “keto flu,” typically occur during the early adaptation phase and generally resolve within several days or weeks. However, long-term ketogenic interventions have also been associated with potential hepatic, renal, and skeletal complications in specific clinical populations [140]. Careful patient selection, nutritional planning, and medical supervision are therefore recommended when ketogenic dietary therapies are implemented. In addition, considerable interindividual variability in lipid responses has been observed during ketogenic dietary interventions. While many individuals experience reductions in triglycerides and increases in HDL cholesterol, elevations in LDL cholesterol or apolipoprotein B may occur in certain cases, particularly when dietary fat quality is suboptimal. Furthermore, in older adults or individuals experiencing rapid weight loss, inadequate protein intake and unintended lean mass reduction may increase the risk of sarcopenia. For these reasons, periodic monitoring of lipid profiles, metabolic parameters, and nutritional status is recommended when ketogenic dietary therapies are implemented in clinical or preventive settings. In addition, potential interactions with commonly used medications should be considered. In particular, glucose-lowering therapies and antihypertensive medications may require adjustment during ketogenic dietary interventions due to changes in glycemic control, body weight, and blood pressure. Therefore, such dietary interventions should ideally be implemented under appropriate clinical supervision.

### 6.8. Long-Term Safety and Sustainability of Ketogenic Dietary Therapies

Although ketogenic dietary therapies have demonstrated short-term metabolic benefits in several studies, evidence regarding their long-term safety and sustainability remains limited. Most clinical investigations evaluating ketogenic interventions are relatively short in duration, typically ranging from several weeks to several months [29,30,83].

Prolonged adherence to ketogenic dietary patterns may increase the risk of micronutrient deficiencies, gastrointestinal disturbances, increased renal acid load, and alterations in lipid metabolism, particularly when diets are poorly planned or lack adequate dietary diversity [83,104,140]. These considerations are especially relevant for older adults and individuals with pre-existing metabolic or cardiovascular risk factors.

Animal studies have suggested potential effects of ketogenic dietary interventions on lifespan and healthspan under controlled experimental conditions [73,74,75,76]. However, extrapolation of these findings to human populations remains uncertain. Human observational studies emphasize the importance of dietary adherence, long-term feasibility, and substantial interindividual variability in metabolic responses [83].

Consequently, ketogenic dietary therapies are best viewed as targeted nutritional interventions that may be appropriate for specific clinical indications and time-limited therapeutic strategies rather than as universal or lifelong dietary approaches. Individualized assessment, dietary planning, and ongoing monitoring are essential to ensure both safety and effectiveness.

## 7. The Semmelweis Workplace Health Promotion Model Program

Translating mechanistic and clinical findings into real-world health promotion strategies represents a major challenge in nutrition and aging research. Within this context, the Semmelweis Workplace Health Promotion Model Program is presented here as an example of how hypothesis-driven dietary and lifestyle interventions may be implemented within a structured occupational health framework aimed at supporting healthy aging.

The program was developed in response to unfavorable demographic and epidemiological trends in Hungary and operates within the broader Healthy Aging research agenda of Semmelweis University. This research framework integrates preclinical, translational, clinical, and public health investigations with the aim of identifying modifiable determinants of unhealthy aging and evaluating preventive strategies across the life course [77]. Importantly, the program does not aim to validate or promote any specific dietary model—including ketogenic dietary therapies—but rather provides a research infrastructure for evaluating nutrition-related hypotheses in real-world environments.

Within this framework, dietary patterns are examined as potential contributors to metabolic, vascular, and cognitive health, with particular attention to feasibility, long-term adherence, and safety. These considerations are especially relevant when translating dietary strategies—such as low-carbohydrate or ketogenic dietary approaches—from controlled experimental conditions to workplace and community settings. Accordingly, the program enables observational and implementation-oriented assessment of how dietary patterns associated with changes in insulin sensitivity, inflammatory status, and vascular function may relate to markers of cerebrovascular health and brain aging at the population level.

The Semmelweis Workplace Health Promotion Model Program should therefore be interpreted primarily as an implementation and feasibility platform, rather than as evidence supporting the efficacy of any specific dietary intervention. In the context of the present review, its relevance lies in illustrating how mechanistic insights related to ketogenic metabolism, vascular function, and neuroinflammatory pathways may be translated into hypothesis-generating, ethically supervised, population-based research frameworks focused on healthy aging [78].

## 8. Discussion

This narrative review integrates current evidence linking ketogenic metabolism and dietary interventions to cerebrovascular regulation and neuroprotective mechanisms relevant to brain aging. Rather than promoting ketogenic dietary strategies as universal interventions, the available literature supports several biologically plausible pathways—including cerebral energy metabolism, mitochondrial function, oxidative stress modulation, and inflammatory signaling—through which nutritional ketosis may influence brain health within an aging vascular–metabolic context.

Experimental studies consistently demonstrate that ketone bodies, particularly β-hydroxybutyrate, can serve as alternative cerebral energy substrates when glucose utilization is impaired. Preclinical models suggest that ketone-based metabolism may enhance mitochondrial efficiency, reduce oxidative stress, and stabilize neuronal bioenergetics under conditions associated with aging and neurodegeneration. In addition, ketone bodies appear to modulate inflammatory signaling pathways, including NF-κB–related mechanisms, potentially contributing to attenuation of chronic neuroinflammation.

From a cerebrovascular perspective, ketogenic dietary interventions may also exert indirect effects on brain health through improvements in metabolic parameters such as insulin sensitivity, glycemic variability, and endothelial function. Because metabolic dysfunction, vascular pathology, and cognitive decline are closely interconnected, these changes may influence processes including neurovascular unit integrity, blood–brain barrier stability, cerebrovascular reactivity, and neurovascular coupling. However, an important limitation in the current literature is the difficulty in distinguishing ketosis-dependent biological effects from broader metabolic improvements associated with weight loss, caloric restriction, or improved dietary quality. Notably, several metabolic benefits reported during ketogenic interventions have also been observed in other dietary strategies, including calorie-restricted and Mediterranean dietary patterns, suggesting that some favorable outcomes may reflect general metabolic improvements rather than ketosis-specific mechanisms alone.

### Ketosis-Dependent Versus Non-Specific Metabolic Effects

A key methodological challenge in evaluating ketogenic dietary interventions is distinguishing ketosis-specific effects from broader metabolic changes accompanying dietary modification. Many clinical studies report improvements in glycemic control, body weight, inflammatory markers, and metabolic risk factors during ketogenic interventions [39,43,152,153,154]. These outcomes may arise from multiple mechanisms—including caloric restriction, weight loss, improved dietary adherence, reduced consumption of ultra-processed foods, or altered macronutrient composition—rather than from ketosis itself.

Mechanistically, ketosis-specific effects are largely mediated by circulating ketone bodies, particularly β-hydroxybutyrate, which may influence mitochondrial efficiency, oxidative stress regulation, and inflammatory signaling pathways [155]. β-hydroxybutyrate has also been proposed to function as a metabolic signaling molecule capable of modulating transcriptional regulation and cellular stress responses, potentially contributing to the neuroprotective and metabolic effects hypothesized in ketogenic dietary therapies [156,157]. In contrast, reductions in insulin resistance, visceral adiposity, and systemic inflammation may occur independently of sustained ketone production and have been documented across multiple dietary interventions achieving comparable weight loss [158,159,160]. Consequently, careful interpretation of the literature requires distinguishing ketosis-dependent mechanisms from broader metabolic adaptations associated with dietary modification [161,162]. Future studies should therefore systematically report circulating ketone concentrations and differentiate ketosis-mediated from weight-loss-mediated effects when evaluating clinical outcomes of ketogenic dietary therapies.

Despite these mechanistic hypotheses, the overall strength of human evidence remains limited. Much of the available literature derives from preclinical experiments or short-term clinical studies characterized by small sample sizes, heterogeneous intervention protocols, and reliance on surrogate metabolic endpoints. Robust evidence regarding clinically meaningful cerebrovascular outcomes—such as stroke incidence, progression of cerebral small-vessel disease, or long-term trajectories of cognitive decline—remains scarce. In this context, translational research frameworks that enable hypothesis-driven evaluation of dietary strategies under real-world conditions may be particularly valuable. The Semmelweis Workplace Health Promotion Model Program is therefore presented as an example of such a translational platform rather than as evidence supporting a specific dietary paradigm. By embedding nutrition-based interventions within occupational and population-level healthy aging initiatives, such frameworks may help bridge mechanistic research and implementation science without presupposing the superiority of any single dietary approach.

## 9. Strengths and Limitations

A key strength of this review lies in its mechanistic focus on cerebrovascular and neuroprotective pathways linking ketogenic metabolism to brain aging. By emphasizing neurovascular regulation, mitochondrial function, and inflammatory signaling rather than broad metabolic outcomes alone, the review aims to provide a focused integrative perspective. The explicit differentiation between preclinical and human evidence, together with the consideration of translational research frameworks, further supports interpretability.

Several limitations should also be acknowledged. The narrative review design inherently carries the potential for selection and publication bias, with positive findings more likely to be reported in the literature. In addition, substantial heterogeneity across studies—including differences in dietary composition, intervention duration, degree of ketosis, and participant characteristics—limits comparability between investigations. Adherence to ketogenic dietary therapies is variably reported and often declines over time, while objective biomarkers of compliance, such as circulating β-hydroxybutyrate concentrations, are inconsistently assessed. Furthermore, many studies rely on surrogate metabolic outcomes rather than clinically meaningful cerebrovascular endpoints. The limited availability of long-term randomized controlled trials examining stroke incidence, neurovascular function, or progression of neurodegenerative disease further constrains causal interpretation. Finally, extrapolation from animal models to human aging and vascular disease remains inherently uncertain, underscoring the need for carefully designed long-term human studies.

## 10. Conclusions

Ketogenic dietary therapies represent metabolically distinct dietary approaches with potential implications for cerebrovascular regulation and neuroprotection. Current evidence—derived predominantly from preclinical research and short-term human studies—suggests that nutritional ketosis may influence brain aging through interconnected effects on cerebral energy metabolism, mitochondrial function, oxidative stress, and inflammatory signaling pathways. Indirect improvements in vascular and cardiometabolic risk factors may further contribute to these associations.

However, evidence supporting the long-term efficacy, safety, and sustainability of ketogenic dietary interventions in human populations remains limited. Heterogeneous study designs, relatively short intervention periods, and variable adherence complicate interpretation of the available literature. Translational research frameworks, such as the Semmelweis Workplace Health Promotion Model Program, may help facilitate hypothesis-driven evaluation of nutrition-based interventions in real-world contexts without endorsing a single dietary strategy.

Overall, ketogenic dietary therapies may offer targeted, context-dependent metabolic benefits, but they should not be interpreted as universal or lifelong dietary solutions. Future research should prioritize well-designed long-term randomized trials, real-world cohort studies, and mechanistic investigations incorporating clinically meaningful cerebrovascular and cognitive endpoints. Until such data become available, ketogenic dietary strategies should be applied cautiously and within individualized, medically supervised frameworks.

## Figures and Tables

**Table 1:** Mechanistic relevance of major ketogenic dietary therapy variants in relation to cerebrovascular and neuroprotective mechanisms.

Ketogenic Variant	Ketone Induction Profile	Insulin Modulation	Neurovascular/Neuroprotective Relevance	Translational Considerations
Classic ketogenic diet (CKD)	Sustained moderate–high ketosis	Strong insulin suppression	Supports alternative cerebral energy metabolism; reduces glucose dependence	Limited long-term adherence
MCT-based ketogenic diet	Rapid and robust ketone production	Moderate insulin suppression	Enhanced cerebral ketone availability; potential relevance for brain energy metabolism	Better tolerability at moderately higher carbohydrate intake
Modified Atkins diet (MAD)	Low–moderate stable ketosis	Mild insulin modulation	Potential metabolic and anti-inflammatory effects with improved adherence	Suitable for longer-term implementation
Low glycemic index treatment (LGIT)	Minimal ketosis	Mild insulin modulation	Indirect neurovascular benefits via glycemic stability	Limited direct ketone-mediated effects
Very low-energy ketogenic therapy (VLEKT)	Mild–moderate nutritional ketosis	Strong insulin suppression	Rapid metabolic effects primarily related to weight loss	Not suitable for long-term use or frail populations

References: [21,22,23,24,25,26,27,28,29,30,31,32,33].

**Table 2:** Human studies and systematic reviews investigating ketogenic dietary therapies and cognitive or neurological outcomes.

Study	Population	Intervention	Duration	Ketosis Verification	Main Outcomes	Limitations
Reger et al., 2004 [62]	Mild–moderate AD and MCI (*n* = 20)	Oral MCT supplementation	Single administration	Plasma β-hydroxybutyrate measured	Improved memory performance (ADAS-cog, paragraph recall) in APOE-ε4− subjects	Small sample size; acute intervention
VanItallie et al., 2005 [65]	Parkinson’s disease patients (*n* = 7 enrolled; *n* = 5 completed)	Hyperketogenic diet (≈90% fat, 2% carbohydrate)	28 days	Serum and urinary ketones measured	Improvement in Unified Parkinson’s Disease Rating Scale (UPDRS) scores (~43% mean reduction)	Small sample size; open-label design; no control group
Cunnane et al., 2021 [63]	Mild cognitive impairment (BENEFIC trial participants)	Ketogenic medium-chain triglyceride (kMCT) nutritional supplement	6 months	Elevated plasma ketone concentrations and increased brain ketone uptake	Improved cognitive performance (free and cued recall, verbal fluency, Boston Naming Test)	Evidence derived from RCT summary; relatively moderate sample size
Bohnen et al., 2023 [60]	Clinical trials in MCI, Alzheimer’s disease, and Parkinson’s disease	Systematic review of ketogenic interventions (diet and MCT supplementation)	Various	Multiple studies measured circulating β-hydroxybutyrate or brain ketone uptake	Evidence suggests probable cognitive benefit in MCI and APOE-ε4− AD patients	Heterogeneity of interventions; limited number of trials
Avgerinos et al., 2020 [61]	Patients with mild cognitive impairment and Alzheimer’s disease (12 human studies; *n* = 422)	Medium-chain triglyceride (MCT) supplementation	Various (acute–6 months)	Increased plasma β-hydroxybutyrate concentrations	Meta-analysis showed mild ketosis induction and modest improvement in cognitive measures (ADAS-Cog/MMSE combined outcomes)	Heterogeneity of included studies; moderate risk of bias; limited long-term trials
Henderson et al., 2009 [67]	Mild–moderate AD (*n* = 152)	Oral AC-1202 (MCT-based ketogenic compound)	90 days	Serum β-hydroxybutyrate measured	Modest improvement in ADAS-Cog scores, mainly in APOE-ε4− subjects	Short duration; genotype-dependent effect; industry-sponsored
Krikorian et al., 2012 [68]	Mild cognitive impairment (*n* = 23)	Very low-carbohydrate ketogenic diet vs. high-carbohydrate diet	6 weeks	Urinary ketones measured	Improved verbal memory performance; ketone levels correlated with memory	Small sample size; short intervention duration
Fortier et al., 2021 [69]	Mild cognitive impairment (*n* = 83 completed)	Ketogenic MCT drink (30 g/day) vs. placebo	6 months	Plasma β-hydroxybutyrate and acetoacetate measured	Improved episodic memory, executive function, and language; cognitive improvements correlated with plasma ketone levels	Moderate sample size; gastrointestinal adverse events; supplement-based intervention

Abbreviations: AD, Alzheimer’s disease; MCI, mild cognitive impairment; MCT, medium-chain triglyceride; kMCT, ketogenic medium-chain triglyceride; RCT, randomized controlled trial; UPDRS, Unified Parkinson’s Disease Rating Scale; ADAS-Cog, Alzheimer’s Disease Assessment Scale–Cognitive Subscale; MMSE, Mini-Mental State Examination; APOE, apolipoprotein E.

## Data Availability

Data sharing is not applicable to this article as no new data were created or analyzed in this study. The authors used ChatGPT 5.0 (OpenAI) solely for language editing and refinement. All content was critically reviewed and approved by the authors, who take full responsibility for the final manuscript.

## Abbreviations

4-HNE	4-Hydroxynonenal
ApoA1	Apolipoprotein A1
ApoB	Apolipoprotein B
BDNF	Brain-Derived Neurotrophic Factor
BHB	β-Hydroxybutyrate
BMI	Body Mass Index
CHD	Coronary Heart Disease
CKD	Classic Ketogenic Diet
CNS	Central Nervous System
GI	Glycemic Index
GL	Glycemic Load
HbA1c	Glycated Hemoglobin
HDL	High-Density Lipoprotein
KDTs	Ketogenic Dietary Therapies
LDL	Low-Density Lipoprotein
LGIT	Low Glycemic Index Treatment
MAD	Modified Atkins Diet
MCT	Medium-Chain Triglyceride
MD	Mediterranean Diet
RCT	Randomized Controlled Trial
sdLDL	Small Dense Low-Density Lipoprotein
TC	Total Cholesterol
VLEKT	Very Low-Energy Ketogenic Therapy
WHO	World Health Organization
